# Automatic Online Motor Control Is Intact in Parkinson’s Disease With and Without Perceptual Awareness

**DOI:** 10.1523/ENEURO.0215-17.2017

**Published:** 2017-10-16

**Authors:** Kate E. Merritt, Ken N. Seergobin, Daniel A. Mendonça, Mary E. Jenkins, Melvyn A. Goodale, Penny A. MacDonald

**Affiliations:** 1 The Brain and Mind Institute, the University of Western Ontario, London, Ontario Canada; 2 Department of Clinical Neurological Sciences, London Health Sciences Centre, University Hospital, 339 Windermere Road, London, Ontario N6A 5A5, Canada; 3 Department of Psychology, the University of Western Ontario, London, Ontario Canada

**Keywords:** Basal ganglia, bradykinesia, dopaminergic therapy, online motor control, parkinson’s disease

## Abstract

In the double-step paradigm, healthy human participants automatically correct reaching movements when targets are displaced. Motor deficits are prominent in Parkinson’s disease (PD) patients. In the lone investigation of online motor correction in PD using the double-step task, a recent study found that PD patients performed unconscious adjustments appropriately but seemed impaired for consciously-perceived modifications. Conscious perception of target movement was achieved by linking displacement to movement onset. PD-related bradykinesia disproportionately prolonged preparatory phases for movements to original target locations for patients, potentially accounting for deficits. Eliminating this confound in a double-step task, we evaluated the effect of conscious awareness of trajectory change on online motor corrections in PD. On and off dopaminergic therapy, PD patients (*n* = 14) and healthy controls (*n* = 14) reached to peripheral visual targets that remained stationary or unexpectedly moved during an initial saccade. Saccade latencies in PD are comparable to controls’. Hence, target displacements occurred at equal times across groups. Target jump size affected conscious awareness, confirmed in an independent target displacement judgment task. Small jumps were subliminal, but large target displacements were consciously perceived. Contrary to the previous result, PD patients performed online motor corrections normally and automatically, irrespective of conscious perception. Patients evidenced equivalent movement durations for jump and stay trials, and trajectories for patients and controls were identical, irrespective of conscious perception. Dopaminergic therapy had no effect on performance. In summary, online motor control is intact in PD, unaffected by conscious perceptual awareness. The basal ganglia are not implicated in online corrective responses.

## Significance Statement

We directly investigated (a) the ability of PD patients to perform online motor corrections and (b) whether these corrections are affected by conscious awareness of target displacements. Contrary to a previously published report by Desmurget et al. (2004), we found that after controlling for the confounding effects of PD-related bradykinesia, automatic, in-flight motor control is intact in PD patients, unaffected by conscious awareness. Further, dopaminergic therapy had no effect on these smooth, in-flight corrections. Despite prominent motor symptoms, our findings suggest that PD patients have intact automatic online motor control. Our results further imply that the striatum and basal ganglia do not mediate online motor corrections.

## Introduction

Parkinson’s disease (PD) disrupts motor functions, especially movement preprogramming (Harrington and Haaland, 1991; Fattapposta et al., 2002). Surprisingly little is known about automatic control of ongoing actions in PD, however. In the double-step paradigm, a target location is specified twice—once before and once during or after an initial orienting saccade. Using this paradigm, Desmurget et al. (2004) found that PD patients adjusted their hand trajectories normally in response to small (4-cm), target location displacements, when the displacement arose during the initial saccade and was thus subliminal (experiment 1). In contrast to performance of healthy age-matched controls, PD patients failed to modify their ongoing trajectories when a target’s location was perturbed 6 cm, at hand movement onset, and hence when displacement was consciously perceived (experiment 2). In summary, PD patients performed small, unconscious modulations of ongoing movement appropriately but evidenced deficits in generating large, consciously-perceived automatic, corrective responses. Desmurget et al. (2004) specifically attributed the difference in PD patients’ performance across experiments 1 and 2 to an impairment in executing online corrections that were consciously perceived. According to these results, the basal ganglia may act as a “motor gate,” controlling the timing and necessity of motor corrections. That is, the basal ganglia may be recruited for “premovement” decisions and feed-forward modeling (Houk et al., 2007; Tunik et al., 2009).

However, the finding of impaired automatic processing in PD directly contradicts the prevailing view that the dorsal striatum (DS), the region most depleted of dopamine in PD, mediates deliberation and the suppression of inappropriate automatic responses (Balleine et al., 2007; MacDonald et al., 2011; Hiebert et al., 2014). Dysfunction of the DS produces a shift favoring more automatic responding (Benke et al., 2003; Rieger et al., 2003; Cameron et al., 2010; Cools et al., 2010). For example, in both Stroop (Henik et al., 1993; Dujardin et al., 1999) and antisaccade (Kitagawa et al., 1994; Briand et al., 1999) tasks, PD patients exhibit a stronger tendency than controls to perform more automatic responses (i.e., word reading and prosaccade movements). Notably, in these tasks, the visual cues are consciously perceived, casting doubt on the conclusion that conscious perception interferes with automatic motor corrections in PD (Desmurget et al., 2004).

On closer examination, aspects of the experimental setup in Desmurget et al. (2004), unrelated to conscious perception, might have differentially impacted PD patients’ performance relative to that of controls. Though saccade onset is normal (Briand et al., 1999; Chan et al., 2005), slowed limb movement onset is a cardinal motor symptom of PD (Berardelli et al., 2001; Klockgether, 2004). Consequently, when target perturbations occurred at limb movement onset in experiment 2, target displacements arose later for patients than for controls. PD patients therefore had more time to prepare their movement toward the initial target position than controls in experiment 2 but not in experiment 1, when target jump was intrasaccadic. Increased preparatory phases for preliminary actions are problematic, because longer preparatory phases make modifying or inhibiting actions more challenging (Lappin and Eriksen, 1966; Logan, 1981). Consequently, PD symptoms translated to a greater challenge adapting to target displacements in experiment 2 relative to controls. Because of this confound and the surprising fact that no similar studies have been performed, the effect of PD on online motor control remains unclear.

Despite the prominence of motor symptoms in PD, the effect of PD on online motor control has received little attention. This was the general aim of the present study. Specifically, we intended to investigate the effect of awareness of a target displacement on online motor correction in PD, avoiding the confounding effect of PD-related bradykinesia. We contrasted large (7-cm) relative to small (3.5-cm) intrasaccadic target displacements. Large target perturbations gain conscious awareness even when they arise during initial fixation-to-target saccade (Bridgeman et al., 1975). In a separate block of trials, we confirmed that this manipulation was effective, explicitly assessing participants’ awareness of target displacement for large versus small jumps. We also investigated the effect of dopaminergic therapy on online motor corrections. This issue has not previously been explored.

## Materials and Methods

### Subjects

Fourteen patients with clinically diagnosed idiopathic PD (4 females and 10 males) and 14 healthy age-matched controls (9 females and 5 males) participated in the study. All participants provided written informed consent according to the Declaration of Helsinki (1991). All procedures were approved by the Health Sciences Research Ethics Board of the University of Western Ontario (London, Ontario, Canada). Participants did not have previous experience with the task and were naive to the purpose of the experiment. All participants were right-handed and had normal or corrected-to-normal vision.

Patients with PD were all diagnosed by a neurologist, levodopa responsive, and taking regular dopaminergic medication. The daily levodopa equivalent dose (mean = 637.77 mg, SD = 370.15) was calculated in accordance with Evans et al. (2004): levodopa dose + levodopa × 1/3 if on entacapone + bromocriptine (mg) × 10 + cabergoline or pramipexole (mg) × 67 + ropinirole (mg) × 20 + pergolide (mg) × 100 + apomorphine (mg) × 8. Patients had no coexisting dementia or other neurologic illness, suspicion of familial PD, or treatment with deep brain stimulation. They were not taking any cognitive-enhancing medications. Control participants had no neurologic or psychiatric illness. They were not taking dopaminergic therapy or cognitive-enhancing medications. There were no statistically significant demographic differences between patients and controls. Participant demographics are presented in Table 1.

**Table 1.** Demographic and clinical information and screening cognitive and affective measures for participants with PD and controls

Group	*n*	Age (y)	Education (y)	Diagnosis duration (y)	Levodopa dose (mg equivalent)	UPDRS score	ANART score	BDI-II score	BAI score	Apathy score	MOCA score
Day 1											
PD	14	65.21 (2.33)	15.79 (0.86)	6.22 (1.32)	637.77 (98.92)	—	—	10.57 (1.17)	9.21 (1.53)	11.57 (1.33)	—
On	7	62.86 (3.64)	15.57 (1.39)	6.86 (1.61)	620.32 (106.93)	8.78 (1.54)	127.32 (2.31)	8.71 (1.77)	6.00 (1.18)	8.86 (0.70)	27.57 (0.61)
Off	7	67.57 (2.9)	16.00 (1.11)	5.57 (1.91)	655.21 (158.24)	11.07 (1.32)	—	12.43 (1.31)	12.42 (2.30)	14.28 (2.18)	—
Control	14	64.27 (2.45)	16.13 (0.79)	—	—	—	—	2.29 (0.67)	2.36 (0.84)	9.14(1.16)	—
On	8	63.0 (3.03)	15.89 (0.86)	—	—	0.13 (0.13)	—	2.50 (0.98)	1.75 (0.70)	9.38 (1.64)	—
Off	6	66.17 (3.82)	16.50 (1.67)	—	—	0.00	128.70 (1.30)	2.00 (0.93)	3.17 (2.00)	8.83 (1.76)	28.83 (0.48)
Day 2											
PD	14	65.21 (2.33)	15.79 (0.86)	6.22 (1.32)	637.77 (98.92)	—	—	10.86 (1.49)	7.64 (1.23)	11.71 (1.69)	—
On	7	67.57 (2.9)	16.00 (1.11)	5.57 (1.91)	655.21 (158.24)	10.35 (1.93)	127.54 (1.55)	13.43 (2.42)	8.86 (2.13)	16.14 (2.09)	27.29 (0.36)
Off	7	62.86 (3.64)	15.57 (1.39)	6.86 (1.61)	620.32 (106.93)	11.28 (1.59)	—	8.29 (1.25)	6.43 (1.21)	7.28 (1.22)	—
Control	14	64.27 (2.45)	16.13 (0.79)	—	—	—	—	2.21 (0.51)	2.14 (0.96)	8.57 (1.01)	—
On	6	66.17 (3.82)	16.50 (1.67)	—	—	0.00	—	2.50 (0.62)	3.00 (2.05)	9.33 (1.54)	—
Off	8	63.0 (3.03)	15.89 (0.86)	—	—	0.13 (0.13)	127.10 (1.66)	2.00 (0.80)	1.50 (0.78)	8.00 (1.40)	27.63 (0.50)

Values are presented as group means (SEM). Screening cognitive and affective measures were completed by participants with PD on medication and by healthy controls off medication. All control participants presented with normal neurologic examinations. Session 1 refers to the first day of testing. Session 2 refers to the second day of testing. UPDRS, Unified PD Rating Scale; ANART, National Adult Reading Test IQ Estimation; BDI-II, Beck Depression Inventory II score; BAI, Beck Anxiety Inventory I score; Apathy, Apathy Evaluation Scale score; MoCA, Montreal Cognitive Assessment measured for participants with PD and for matched control participants..

All patients and controls participated in two identical testing sessions on separate days. For PD patients, they were tested once while taking their usual prescribed dopaminergic therapy and once after withdrawal from dopaminergic medication. In the OFF dopamine session, patients were instructed to abstain from all dopaminergic medications including dopamine precursors such as levodopa, aromatic-l-amino-acid decarboxylase inhibitors such as carbidopa, and catechol-*O*-methyltransferase (COMT) inhibitors such as entacapone for a minimum of 12 to a maximum of 18 h, and dopamine agonists, such as pramipexole (Mirapex), ropinirole (Requip), or pergolide (Permax), as well as amantadine (Symmetrel), rasagiline (Azilect), and selegiline (Eldepryl or Deprenyl) for 16–20 h before testing. Healthy controls received levodopa/carbidopa 100/25 mg (i.e., levocarb) orally in the ON session and cornstarch placebo in the OFF session. Levocarb and placebo were presented in an identical capsule for blinding of participants, each administered 45 min before motor testing. Administering levodopa to healthy controls allowed us to investigate the effects of this medication independent from PD pathology on online motor control. The ON-OFF order was counterbalanced across participants.

A neurologist with subspecialty training in movement disorders evaluated the presence and severity of PD symptoms, when participants were both on and off dopaminergic medication, using the Unified PD Rating Scale (UPDRS) Motor Subscale. Control participants were also assessed using the UPDRS to screen for any undiagnosed neurologic illness. All participants completed a series of standardized cognitive and affective screening tests as well. The mean cognitive and affective screening scores and the UPDRS motor subscale scores appear in Table 1.

### Apparatus and stimuli

**Figure 1.**
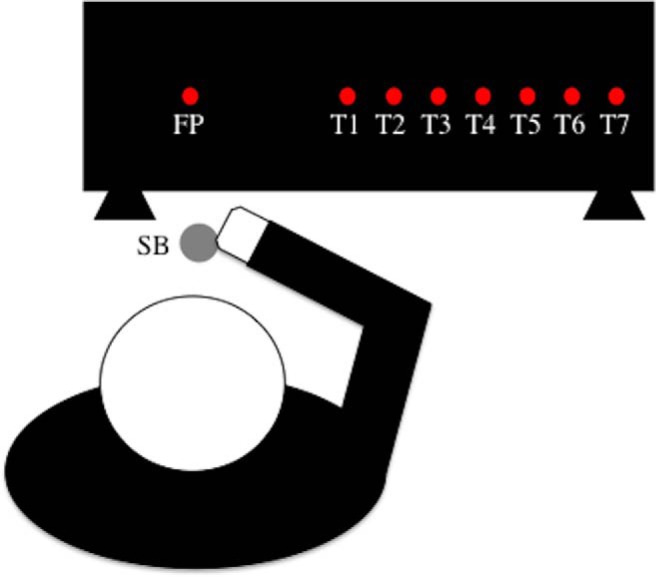
Schematic of experimental setup. The fixation point (FP) and the target lights are represented by red circles. Only one red light was illuminated at a time during the actual experimental procedure. The participant began each trial with their right pointer finger depressed on the start button (SB).

Each participant sat at a table with head stabilized in a chin-rest. All tasks were performed in a darkened room to minimize the effect of spatial cues and visual feedback of the pointing hand. A pressure-sensitive start button was fastened to the table directly in front of the participant and ∼10 cm from the edge of the tabletop. The stimuli were presented on a vertically mounted, custom-built display board that consisted of a horizontal array of red light-emitting diodes (LEDs) set below a transparent Plexiglas surface. Each LED was 5 mm in diameter. The board was secured to the table such that the leftmost LED, which functioned as the fixation point, was positioned 40 cm forward from the subject’s midline and aligned with the start button. All other LEDs served as targets and were horizontally aligned at 7 distances to the right of the fixation point: 24.5, 28, 31.5, 35, 38.5, 42, and 45.5 cm (Fig. 1). These targets are referred to as T1–T7, respectively.

Infrared LEDs (IREDs) were attached to the participant’s right index finger and inner wrist with adhesive tape. The experimenter ensured that the pad of the participant’s index finger was unobstructed. The diode wires were secured to permit unrestricted arm movements. The 3D positions of the IREDs were recorded with an optoelectronic motion capture system, Optotrak Certus (Northern Digital) at 200 Hz. Monocular eye position was recorded at 1000 Hz with the Eyelink 1000 table-mount eye-tracking system (SR Research).

### Procedure

Experimental procedures were identical in sessions 1 and 2. Participants performed a reaching and a target displacement judgment task. In the reaching task, participants were instructed to point to a peripheral visual target that either remained stationary (stay condition) or unexpectedly changed locations (jump conditions). On jump trials, unexpected location changes occurred during the initial saccade from the central fixation to the peripheral visual target. In this way, the target jump was not linked to limb movement onset. In the small jump condition, the displacement from central fixation was 3.5 cm to the right or left of the initial peripheral target location. In the large jump condition, the displacement was 7 cm. The size of the target displacement was expected to affect conscious awareness of the jump. In the small jump condition, we intended to induce online motor corrections that were not consciously perceived. In the large jump condition, we expected automatic motor corrections that were consciously perceived. To confirm that this method was effective, participants performed a two-alternative forced-choice target displacement judgment task. In this task, they explicitly indicated their conscious awareness of target displacements. Both small and large target displacements were assessed in random order, paired on each trial with a stay display.

For both the reaching and target displacement judgment tasks, participants began by staring at a central fixation point. As soon as the fixation point was extinguished, an LED light (target) was illuminated at one of seven peripheral locations (T1–T7). Participants were instructed to look toward the target as quickly and as accurately as possible. The target either remained stationary or was unexpectedly displaced by a distance of 3.5 or 7 cm during the participant’s initial orienting saccade. Target displacements were only initiated from either T3 or T5 locations and could occur either to the left or the right of the original target location. The distance between each target location was 3.5 cm, meaning that a small displacement would constitute a jump from T3 to T2, T3 to T4, T5 to T4, or T5 to T6, whereas a large displacement would include those directed from T3 to T1, T3 to T5, T5 to T3, and T5 to T7. Each target jump type, specified by size, direction, and starting position, occurred with equal frequency throughout the experiment. For all statistical comparisons, ANOVAs were considered significant when the *p*-value, corrected for multiple comparisons, was <0.05. The pointing task and the target displacement judgment task differed as follows.

#### Double-step reaching task

**Figure 2.**
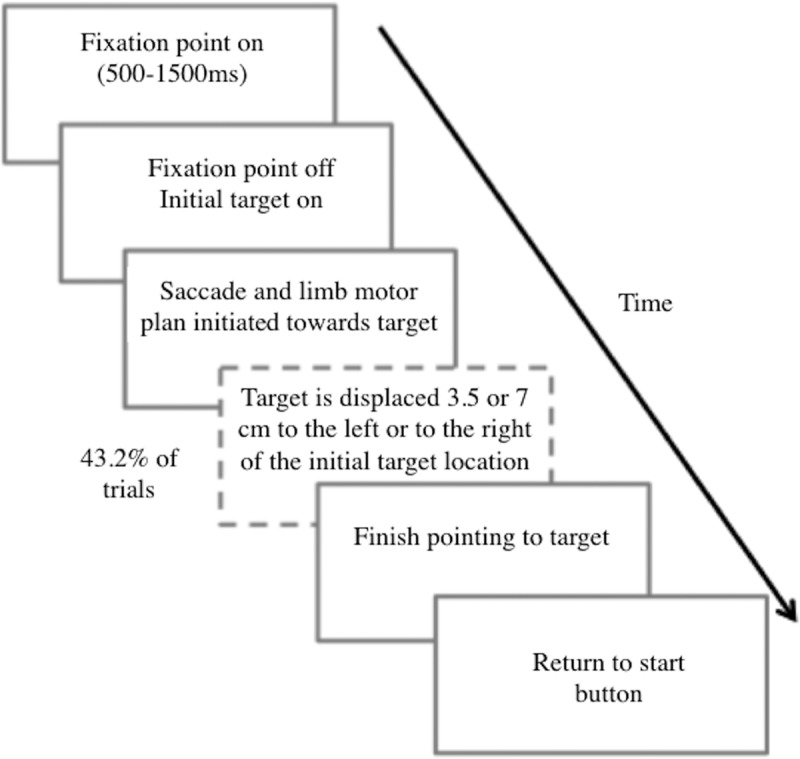
Timeline of trial events. Schematic representation of trial events across time in the double-step pointing task. Adapted from Johnson and Haggard (2002).

Each participant began each trial by depressing a pressure-sensitive start button with the right index finger and staring at the fixation point for 500–1500 ms. On presentation of the peripheral target, participants were instructed to release the start button and to reach for the target as quickly and as accurately as possible. The task consisted of 222 trials. To prevent any predictive behavior, the target remained static in 56.8% of the trials and was displaced in 43.2% of the trials. Further, small jumps were expected to occur without participants’ awareness and hence would be experienced similarly to stay trials. Each stationary condition was presented 18 times, whereas each jump condition occurred 12 times. Jump and stationary trials were randomly interspersed. Trial order was randomized across participants. The target remained visible for the duration of the movement and extinguished when a participant touched it with the pointer finger. On touching the target, participants were instructed to return the pointer finger to the start button to initiate the next trial (Fig. 2).

#### Target displacement judgment task

In each session, participants performed two blocks of the target displacement judgment task, one before and one after the double-step reaching task. Each block consisted of 32 trials. Each trial was composed of a pair of sequential displays. In each pair, a display equivalent to a stationary trial and another equivalent to a jump trial from the reaching task were presented in counterbalanced order. In each block, every jump trial type, specified by jump size, direction, and starting location, was presented two times, for a total of four trials in the experiment. Each stationary trial type was also presented twice per block, with the exception of T4, which was presented four times per block. This was to achieve equal presentations of each of the possible end positions for stay and jump trials. The pairing of stationary and jump displays was randomized. Participants were not required to point to the peripheral target. They simply judged whether display A or B contained a peripheral target that was displaced from its original location. The percentage of correct responses was calculated and compared to chance level.

### Data processing and analyses

#### Double-step reaching task

Analyses were performed in two steps. First, we analyzed eye and hand movements directed toward stationary targets. Second, we evaluated the effect of target displacement on reach kinematics and trajectories. For both steps, the kinematics of each trial were analyzed offline. To isolate the dependent variables, we restricted the data set to include only points during which the hand was in motion in the forward reach trajectory. Thus, we defined the beginning of the movement as the first of five consecutive sample frames in which the wrist IRED exceeded a threshold velocity of 40 mm/s. We defined the end of the movement as the frame with the maximum *y*-spatial coordinate. If a straight line was drawn between the start button and the array of target lights it would represent increasing depth distance (*y* axis). Therefore, the maximum *y*-spatial coordinate correlated to the end position when the full reach distance was achieved (i.e., when the target was touched). The specifics of each analysis are described below.

**Eye movements: stationary targets.** Saccade RT was the dependent variable of interest. We predicated the study on equal saccade RT, and thus timing of target perturbation, across groups. To confirm this, we ran a 2 × 2 repeated-measures ANOVA with *group* as the between-subjects factor (PD versus control) and *dopaminergic medication status* (ON versus OFF) as the within-subject factor.

**Kinematic and reach trajectories: stationary trials.** Hand RT, movement duration (MD), maximum acceleration, and peak velocity were dependent variables extracted from the kinematic data. Hand RT was defined as the time it took to release the start button and initiate reaching after illumination of a peripheral target. MD referred to time from movement onset to reaching the target and movement offset. Separate 2 × 2 mixed ANOVAs, with *group* (PD versus control) as the between-subject factor and *dopaminergic medication status* (ON versus OFF) as the within-subject variable were performed on the four dependent measures.

**Kinematic and reach trajectories: jump trials.** The principal dependent measures extracted to assess online corrections were MD difference scores and points of divergence. MD difference scores were calculated with the following equation: Mean MD Jump Target (A) → Target (B) – Mean MD Stay Target (B). Single sample *t* tests were performed on all MD difference scores for each group, session, and jump size. A 2 × 2 × 2 mixed ANOVA was performed with the between-subjects factor as *group* (PD versus control) and the within-subject variables as *dopaminergic medication status* (ON versus OFF) and *target jump size* (small versus large).

Points of divergence were characterized as the frame at which a reach trajectory on jump trials diverged away from its original hand path to reach the new target location. To determine these points, reach trajectories were first smoothed and normalized in accordance to functional data analysis techniques established by Ramsay and Silverman (2002). The data were normalized such that each trajectory was defined at 300 points equally spaced in the *y* dimension. As such, the continuously defined data curve constituted a single functional observation, rather than its individual discrete data points (Ramsay and Silverman, 2002; Levitin et al., 2007). We conducted a set of planned mixed functional ANOVAs to contrast each jump type with its corresponding stationary condition (either T3 or T5), across the between-subject factor of *group* (PD versus control) and the within-subject variable of *dopaminergic medication status* (ON versus OFF). Functional ANOVAs were performed in Matlab 2014 using customized code adapted from http://www.psych.mcgill.ca/misc/fda/. Functional ANOVAs extend the univariate ANOVA to all points in a trajectory. In this manner, a single functional comparison is performed through the implementation of individual repeated-measures ANOVAs at each frame, as a surrogate for a single statistical comparison of the entire function (Ramsay and Silverman, 2002). We defined initial point of divergence as the point at which >10 consecutive time points for jump trial conditions differed significantly from their respective stationary trial conditions at *p* < 0.05, corrected for multiple comparisons.

#### Target displacement judgment task

To assess perceptual awareness of the target jump, the percentages of correct responses for each group and for each jump size were compared to the chance level (50%) using separate one-sample *t* tests. Further, we ran a 2 × 2 × 2 mixed ANOVA with *group* as the between-subject factor (PD versus control) and *dopaminergic medication status* (ON versus OFF) and *target jump size* (large versus small) as the within-subject factors. The dependent variable was percentage of correct responses.

## Results

### Saccade RT and target jump timing results

**Figure 3.**
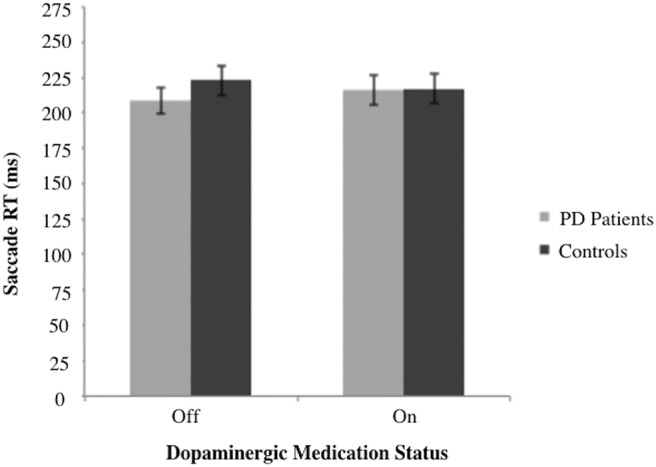
Primary saccade RT in response to initial target appearance. RT is presented as a function of dopaminergic medication status for PD participants (*n* = 14) and matched controls (*n* = 14). The mean values are presented with the error bars reflecting standard error about the mean.

A 2 × 2 mixed ANOVA revealed no main effect of group [*F*(1,26) = 0.259, *MSe* = 799.2, *p* = 0.595] or dopaminergic medication status [*F*(1,26) = 0.068, *MSe* = 13.13 *p* = 0.797] on initial saccade RT. There was also no significant interaction between group and dopaminergic medication status [*F*(1,26) = 3.045, *MSe* = 591.71, *p* =0.093; Fig. 3]. In addition, we directly confirmed that the exact timing of target jumps did not significantly differ between groups [*F*(1,26) = 0.012, *MSe* = 13.17, *p* = 0.913] or across dopaminergic medication status [*F*(1,26) = 2.12, *MSe* = 268.96, *p* = 0.159]. Further, these variables did not interact [*F*(1,26) = 0.774, *MSe* = 98.78, *p* = 0.387]. This confirmed that equal preparatory phases occurred for both groups and across all conditions.

### Limb movement characteristics: stationary trials

**Figure 4.**
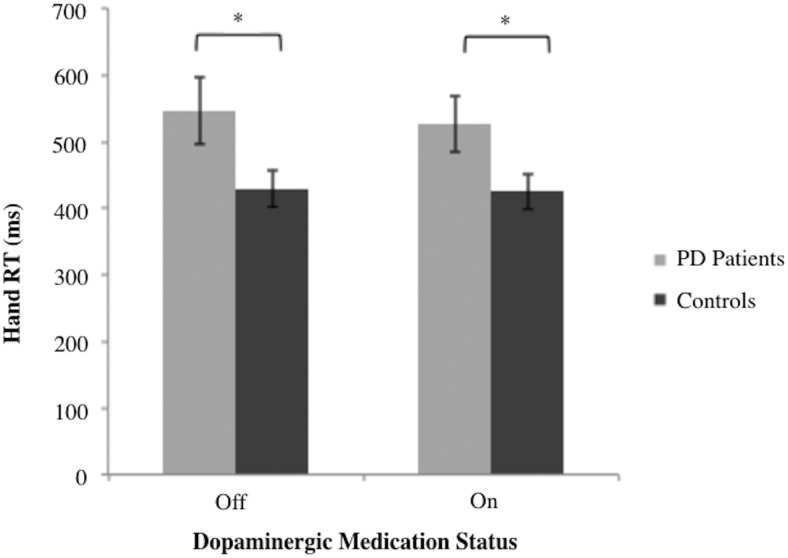
Primary hand RT in response to initial target appearance. RT is presented as a function of dopaminergic medication status for PD participants (*n* = 14) and matched controls (*n* = 14). The mean values are presented with the error bars reflecting standard error about the mean. *p < 0.05.

Patients with PD exhibited significantly longer hand RTs [*F*(1,26) = 4.64, *MSe* = 1.66 × 10^5^, *p* <0.05; Fig. 4] and significantly decreased peak velocities compared with healthy controls [*F*(1,26) = 5.58, *MSe* = 1.31 × 10^6^, *p* < 0.05]. However, there was no significant main effect of group on overall MD [*F*(1,26) = 3.48, *MSe* = 1.10 × 10^6^, *p* = 0.073] or on maximum acceleration [*F*(1,26) = 2.61, *MSe* = 1.57 × 10^8^, *p* = 0.118]. Dopaminergic medication status did not significantly affect any of the dependent variables including hand RT, MD, peak velocity, or maximum acceleration, all *F* < 1. The group × dopaminergic medication status interaction was not significant for any of the dependent variables [*F*(1,26) = 0.174, *MSe* = 761.1, *p* = 0.68 for hand RT; *F*(1,26) = 0.009, *MSe* = 405, *p* = 0.926 for MD; *F*(1,26) = 2.859, *MSe* = 6.55 × 10^4^, *p* = 0.103 for peak velocity; *F*(1,26) = 2.40, *MSe* = 4.83 × 10^7^, *p* = 0.133 for maximum acceleration].

### Limb movement characteristics: jump trials

**Figure 5.**
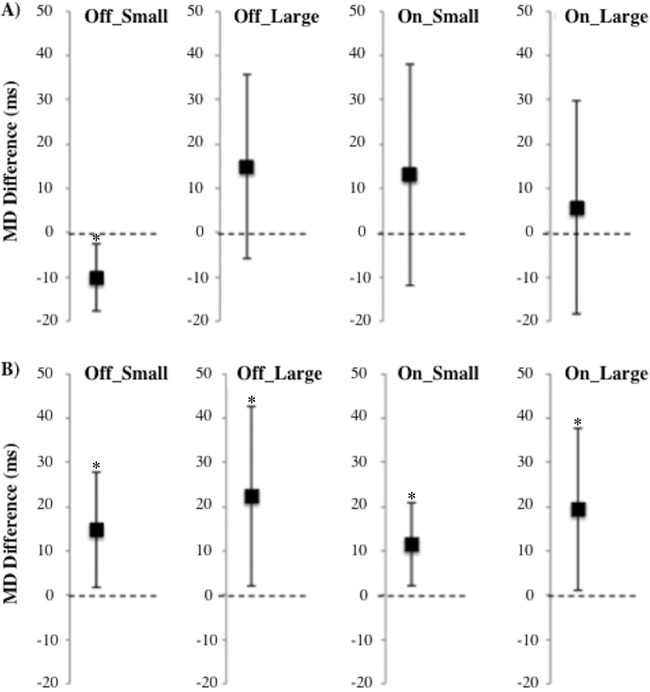
Movement duration (MD) difference scores compared to zero. (A) PD patients (*n* = 14). (B) Controls (*n* = 14). MD differences are displayed for each medication status and target jump size. Participants performed the task in either the ON-OFF or OFF-ON medication orders. The error bars reflect a 95% confidence interval. MD difference scores are significantly above 0 for healthy controls but not PD patients. *p < 0.05.

MD difference scores for jump trials minus stationary trials were not significantly greater than zero for PD group across any of the condition types [*t*(13) = 1.543, *p* = 0.147 for PD OFF large; *t*(13) = –2.915, *p* = 0.012 for PD OFF small; *t*(13) = 0.509, *p* = 0.620 for PD ON large; *t*(13) = 1.128, *p* = 0.280 for PD ON small], indicating that online corrections were automatic (Fig. 5*A*). Patients had significantly shorter MDs when reaching in trials with small target jumps relative to their respective stay trials in the OFF session.

In contrast, controls demonstrated MD difference scores significantly greater than zero across all condition types regardless of their medication status [*t*(13) = 2.38, *p* < 0.05 for controls OFF large; *t*(13) = 2.44, *p* < 0.05 for controls OFF small; *t*(13) = 2.289, *p* < 0.05 for controls ON large; *t*(13) = 2.654, *p* < 0.05 for controls ON small; Fig. 5*B*].

The mixed ANOVA revealed a trend toward larger MD difference scores for healthy controls relative to PD patients [*F*(1,26) = 3.988, *MSe* = 3.47 × 10^3^, *p* = 0.056], indicating a slight cost for controls but not for PD in amending their reach trajectories from an original to a final target locations. Neither the main effect of dopaminergic medication status [*F*(1,26) = 0.123, *MSe* = 107.55, *p* = 0.729] nor the effect of target jump size [*F*(1,26) = 1.369, *MSe* = 1.94 × 10^3^, *p* = 0.253] was significant. The latter finding indicates that regardless of whether target displacements were consciously or unconsciously perceived (i.e., large or small target displacements, respectively), online motor corrections were performed equivalently. There were no significant two- or three-way interactions between group, dopaminergic medication status, and target jump size, all *F* < 1.

**Figure 6.**
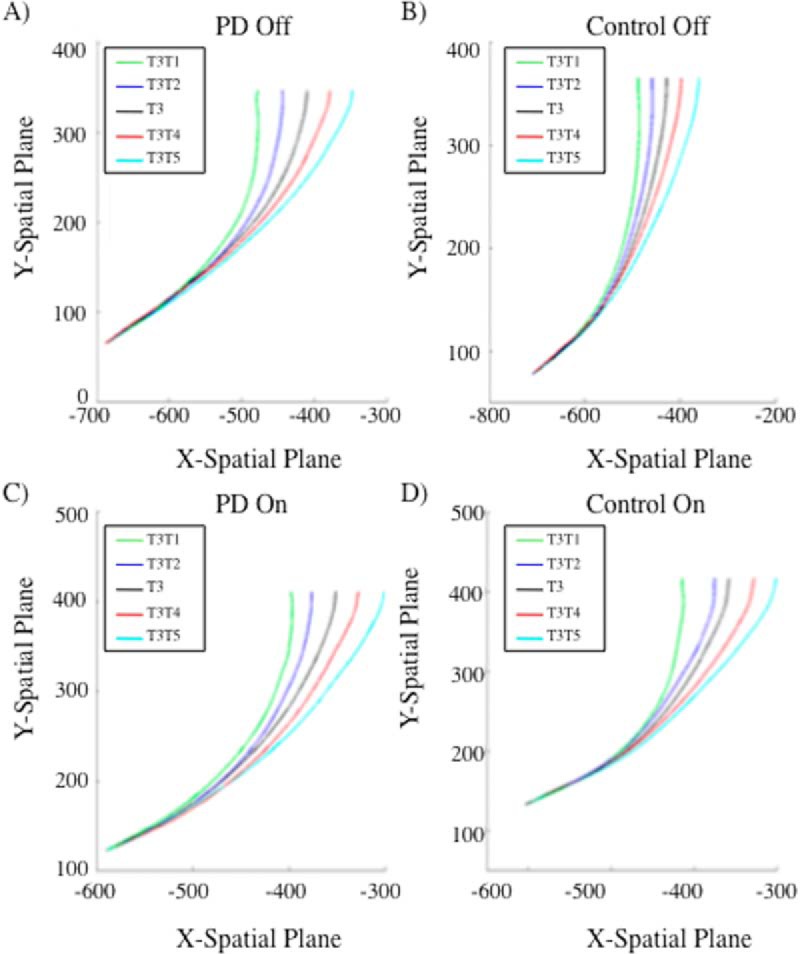
Mean trajectory plots for reaches originally directed to T3 for (A) PD patients off dopaminergic medication, (B) controls off dopaminergic medication, (C) PD patients on dopaminergic medication, and (D) controls on dopaminergic medication. Black line represents the baseline reach to stationary T3. PD patients do not significantly differ from controls at the point of divergence for any of the reach comparisons both on and off of dopaminergic therapy.

**Figure 7.**
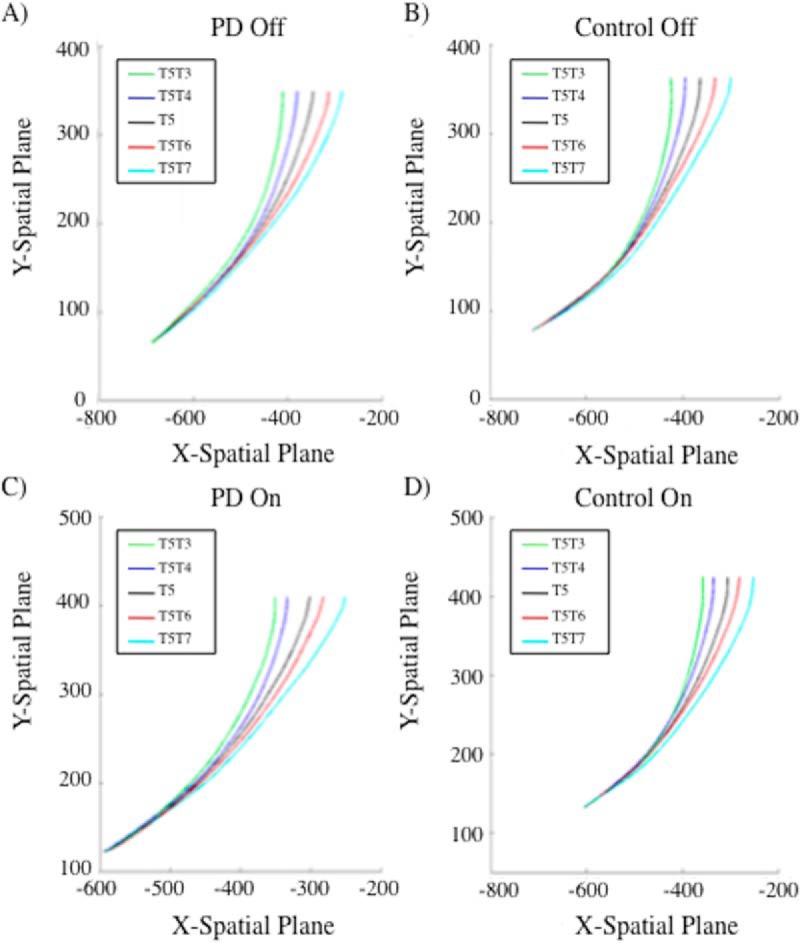
Mean trajectory plots for reaches originally directed to T5 for (A) PD patients off dopaminergic medication, (B) controls off dopaminergic medication, (C) PD patients on dopaminergic medication, and (D) controls on dopaminergic medication. Black line represents the baseline reach to stationary T5. PD patients do not significantly differ from controls at the point of divergence for any of the reach comparisons both on and off of dopaminergic therapy.

As illustrated in Figs. 6 and 7, target end-position had a significant effect on lateral deviation throughout the reach in both groups, unaffected by dopaminergic therapy. We implemented 2 × 2 × 2 mixed-measures functional ANOVAs to assess pairwise comparisons between jump trials and their relative stay trials across the movement trajectories. *Group* was the between-subject factor (PD versus control), whereas *dopaminergic medication status* (ON versus OFF) and *condition* (jump versus stay) were within-subject variables. A main effect of condition (jump versus stay) revealed that the trajectories for jump trials significantly diverged from that of stay trials after following a similar course for a percentage of the trajectory. There were no significant effects of group or dopaminergic medication status in terms of onset or degree of divergence. There were no significant two- or three-way interactions between group, dopaminergic medication status, or condition.

Half of our jump trials were initiated from T3 and half from T5. We report our divergence analyses relative to this preliminary target position, as divergence was based on relative deviations from the original target trajectory path. For trajectories initially directed to T3, large target displacements had a relatively early effect on reach trajectories, such that a smooth divergence was noted at 17% and 13% into the total *y*-movement for T3T1 and T3T5 trials, respectively. Similar results were observed for large displacements for movements initially directed to T5. T5T3 diverged at 18% and T5T7 diverged at 14% into the total *y*-movement. The pairwise functional comparisons of small target displacements revealed a smooth divergence in reach trajectories at 26%, 27%, 31%, and 34% of the total *y*-movement for T3T2, T3T4, T5T4, and T5T6 conditions, respectively. All jump trajectories significantly differed from their relative stay trial in the *x*-dimension from the identified point of divergence onward, until the endpoint of movement. Trajectories appeared to deviate earlier for large relative to small target jumps owing to larger divergence being more apparent and detectable. Group did not interact with condition in any of the functional pairwise comparisons, suggesting that disease status did not significantly affect the ability to diverge trajectories smoothly and at an appropriate time. Dopaminergic medication status significantly interacted with condition for only the T5T6 pairwise comparisons between frames 261 (at 87% of total *y*-movement) and 288 (at 96% of total *y*-movement), for a duration of 9% of the trajectory. All other functional comparisons did not reveal any significant interactions between group and dopaminergic medication status. This indicates that PD diagnosis and medication status did not significantly influence the point at which movements began to diverge or the direction and smoothness of divergence when target location was displaced relative to its initial location. There was not a significant three-way interaction between group, medication status, and condition for any of the functional pairwise comparisons.

### Target displacement judgment task: perceptual awareness results

**Figure 8.**
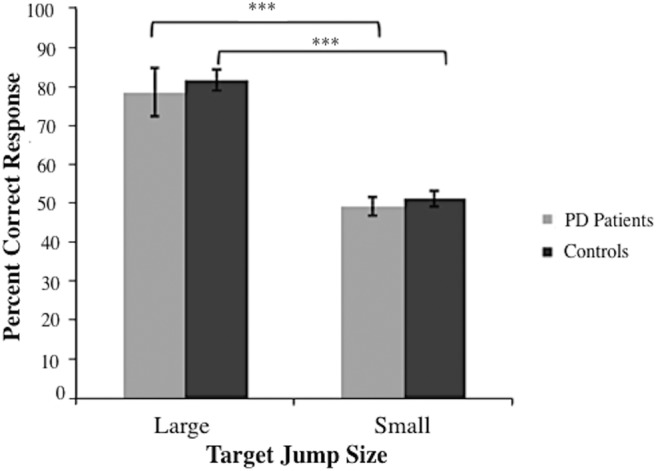
Percentage of correct responses in target jump judgment two-alternative forced choice task. Correct responses are shown as a function of target jump size. Means of the percentage of correct responses are collapsed across medication status for both groups (*n*_PD_ = 14, *n*_control_ = 14). The error bars reflect standard error about the mean. ***p < 0.001.

Target jump size had a significant effect on percentage of correct responses [*F*(1, 26) = 79.60, MSe = 1.24 × 10^4^, *p* < 0.001], with greater accuracy resulting for large [80.1%; accuracy >50% *t*(13) =4.603 *p* < 0.001 for PD; t(13) = 11.746, *p* < 0.001 for controls] relative to small [50.3%; accuracy >50% *t*(13) = –0.240, *p* = 0.814 for PD; *t*(13) = 0.599, *p* = 0.560 for controls] target jumps. This confirmed that the size of the intrasaccadic peripheral target displacement influenced conscious perceptual awareness (Fig. 8). The main effects of group and dopaminergic medication status, and all two- and three-way interactions, were not statistically significant, all *F* < 1. In this way, for both groups and in both sessions, large target jumps were consciously perceived, but small target jumps were not. For small target jumps, correct identification of the jump relative to the stay display in a pair was not different from chance.

## Discussion

We investigated online motor control in PD, specifically the effect of conscious awareness of trajectory corrections on performance in a double-step paradigm. On jump trials, target displacements occurred during an orienting saccade. We found that PD patients and healthy controls had equivalent saccade RTs. Consequently, on jump trials, target displacements arose at comparable times for patients and controls. For both groups, large but not small target jumps were consciously perceived. By explicitly testing this perception in a separate target displacement judgment task, we confirmed that our experimental manipulation had the intended effect. Neither saccade RT nor displacement judgments were affected by dopaminergic therapy in either group. PD patients had longer latencies for limb movement onset, as well as in peak movement velocities compared to controls, as was observed by Desmurget et al. (2004), corroborating the concern that PD patients’ reaching to a target is disproportionately, adversely impacted by procedures that link target perturbations to movement onset. Considering all of these findings, we succeeded in controlling for the confounding effects of PD-related bradykinesia and in designing a study that could directly investigate the effect of conscious perceptual awareness of target displacement on online motor corrections in PD.

We found that MD difference scores for small and large jump relative to stay trials were not significantly greater than zero for PD patients, suggesting that online corrections in response to target displacements were performed automatically. In fact, there was a trend toward lower mean MD difference scores for PD patients compared with healthy controls. Controls evidenced a small cost for trajectory changes in jump trials, discussed below. Trajectory analyses of kinematic data using functional ANOVAs revealed parallel movement trajectories for patients and controls on jump and stay trials, respectively. Onset of divergence and smooth deviation to the new target location on jump relative to stay trials was equivalent for PD patients and their healthy counterparts (see Figs. 6 and 7). Whether or not target displacements were consciously perceived (i.e., respective of jump size), trajectories were the same for PD patients and controls, resolving our central question. Patients and controls performed equivalently respective of jump direction or dopaminergic therapy. Trajectory divergences on jump relative to stay trials corresponded for PD and controls, unaffected by dopaminergic therapy, in eight separate replications (i.e., 2 × 2 × 2 functional ANOVAs). Replications arose due to the inclusion of (a) two different target positions from which displacements could originate, (b) two jump sizes, and (c) two jump directions.

### Effect of conscious awareness on automatic online corrections in PD

Desmurget et al. (2004) found that PD patients and controls performed comparably, amending their trajectories smoothly and automatically when target displacements were small (4 cm) and intrasaccadic. PD patients did not automatically alter movement trajectories when targets were displaced by a larger distance (6 cm) at limb movement onset. Small target displacements that arise during a saccade are subliminal, whereas large target displacements that occur at movement onset are consciously perceived. Desmurget et al. (2004) interpreted their findings in light of these facts and concluded that PD patients are impaired in online corrections when target displacements are consciously perceived.

In their study, however, conscious awareness of the need for trajectory amendments was confounded with target jump trigger: saccadic eye movements or limb movement. Critically, in their experiments 1 and 2, the movement that triggered the target jump was differentially affected by PD. Although saccade latencies are equivalent for patients and controls, limb movements are delayed. In this way, when target displacements were linked to saccadic eye movements in experiment 1, the latency of target displacement was comparable between patients and controls. However, when target jumps were related to limb movement onset in experiment 2, the target displacement was delayed for patients. This prolongation of the period from target onset to target displacement for PD patients relative to controls resulted in a longer preparatory phase for the reaching movement to the initial target location. Movement correction is impacted by the length of the preparatory phase for the original movement. Liu and Todorov (2007) demonstrated that young healthy adults were unable to fully amend their trajectories in response to late-occurring target perturbations (i.e., 300 ms after movement onset). Similarly, delayed corrections have also been observed when targets are displaced at the time of peak movement velocity (Komilis et al., 1993). As a movement plan progresses, the visuomotor system seems to be less efficient at correcting potential errors (Liu and Todorov, 2007; Sarlegna and Mutha, 2015). This provided a plausible alternative interpretation for the findings of Desmurget et al. (2004) and motivated the current experiment.

Here, we directly and unambiguously investigated the effect of conscious awareness of trajectory amendments on online motor control in PD, ensuring equivalent onset of target displacements for (a) consciously-perceived and subliminal target jumps and (b) patients and their age-matched controls. Jump size manipulated conscious awareness. Participants consciously perceived 7-cm, but not 3.5-cm, target displacements. Whether or not target displacement was consciously perceived, patients and controls performed equivalently, clarifying the findings of Desmurget et al. (2004). Furthermore, we suggest that our results are not simply attributable to a lack of statistical power, nor could features of our paradigm render it insensitive to true differences. First, we showed that our experimental paradigm was in fact capable of reliably detecting divergences in trajectories between stay and jump trials. Divergence in reach trajectories became significantly apparent early on in the action, suggesting that our functional data techniques were sensitive to slight changes in position. Second, we used more than double the number of PD patients in our study than were used in the original design of Desmurget et al. (2004). Given that, despite their small sample size, Desmurget et al. (2004) still reported significant differences between healthy controls and PD patients, we have confidence that our experiment was adequately powered. Last and most compelling, we had a total of eight different replications in both the ON medication session and the OFF medication session to find differences between PD and healthy controls if they were indeed present.

### Basal ganglia and dopamine in automatic, online motor control

The finding of equivalent online motor corrections for PD patients compared with age-matched controls, even off dopaminergic therapy, casts substantial doubt on the prospect that the striatum and basal ganglia mediate automatic motor control. This pattern of findings was observed with high reliability in eight separate trajectory analyses. Further, movement durations for target displacement trials were not significantly prolonged relative to stationary target trials in patients, suggesting that correction of movement trajectory is automatic. In PD, the striatum—the DS in particular—is seriously dopamine depleted, and its functions are highly compromised (Bernheimer et al., 1973; Cools 2006; MacDonald and Monchi, 2011). Bolstering the notion that these motor processes are independent of the striatum and basal ganglia, dopaminergic therapy had no effect on smooth modulation of ongoing movement in PD patients. Dopaminergic therapy enhanced other aspects of movement, attested to by significant improvement on the motor subscale of the UPDRS. Even in the OFF state, patients were consistently capable of using feedback online to update their internal representations of goal positions, appropriately amending their actions in-flight. This is consistent with previous research that PD patients successfully use continuous sensory feedback during reaching or tracking movements (Flowers, 1976; Day et al., 1984; Ghilardi et al., 2000). A number of studies support the role of the posterior parietal cortex, a dopamine-independent brain region, in supervising and regulating online context-dependent motor commands (Desmurget et al., 1999; Gréa et al., 2002; Buneo and Andersen, 2006)

In contrast to online motor corrections, reach initiation (i.e., hand movement onset) and movement speed (i.e., peak hand movement velocity) were impaired in PD patients relative to healthy controls. We did not find significant improvements on these measures related to dopaminergic therapy. It is worth noting that the magnitude of improvements induced by exogenous dopamine seems greater with increasing movement complexity, such as when patients execute multiple chained action plans (Benecke et al., 1987; Shook et al., 2005; Hood et al., 2007; Hanna-Pladdy and Heilman, 2010). Because not all movement symptoms are equally affected by dopaminergic therapy, it is plausible that simple, stimulus-driven, reaching movements are among those that are less sensitive.

### Striatum and PD

Online reach corrections are automatic, performed by a reflexive orienting system that seems not disrupted by PD. In fact, movement durations for jump relative to stay trials were not increased in patients though they were for our age-matched control group. There was a trend toward lower movement duration, jump-stay difference scores for patients than controls. This suggests that patients were performing online corrections more efficiently than controls.

Because of DS’s role in promoting deliberation and suppression of automatic behavioral responses, DS dysfunction has been shown to enhance, rather than impair, automatic processing (Benke et al., 2003; Cameron et al., 2010; Cools et al., 2010). In PD, this has translated to heightened automaticity in oculomotor studies using antisaccade versus prosaccade tasks (Praamstra and Plat, 2001; Chan et al., 2005; Fielding et al., 2005), as well as in cognitive assessments such as the Stroop task (Brown and Marsden, 1988; Dujardin et al., 1999; Djamshidian et al., 2011). As an intriguing possibility, aging-related inefficiencies in the online motor control system seemed masked by enhanced automaticity owing to DS deficiency in PD patients.

## Conclusion

Our results support the notion that PD-related bradykinesia prolonged the preparatory phase for patients’ movements to the original target in the design of Desmurget et al. (2004). This rendered smooth modulation of reaching to the new target location more challenging for patients than controls. Here, we controlled for this confound, using the double-step paradigm with large, consciously-perceived and small, subliminal intrasaccadic target displacements. PD patients and controls both performed online motor modifications accurately and equivalently, unaffected by conscious perception of trajectory change. Further, dopaminergic therapy did not influence online motor corrections. Our results support the view that the basal ganglia are not implicated in these corrective responses.

## Synthesis

Reviewing Editor: Kirill Martemyanov, The Scripps Research Institute Decisions are customarily a result of the Reviewing Editor and the peer reviewers coming together and discussing their recommendations until a consensus is reached. When revisions are invited, a fact-based synthesis statement explaining their decision and outlining what is needed to prepare a revision will be listed below. The following reviewer(s) agreed to reveal their identity: Ian Cameron, Pietro Mazzoni.

Both reviewers agreed on the significance and impact of the work presented in the manuscript. They thought that the study presents and important advance in large part because it corrects the view dominated by the previous study in regard to the role of basal ganglia in motor control in Parkinson's disease. It was felt that the findings as presented point to directions of future research on motor control abnormalities in PD that are more in line with the current computational framework of motor control. The reviewers were also satisfied with revisions made in response to review at Journal of Neuroscience. They identified only a few minor correction to take care of for the manuscript to be accepted. Specifically, attention to the following is needed:

1. Typo : page 1, line 10 “Eliminating this confound in a double-step task, we evaluated THE effect of conscious awareness of trajectory change on online motor corrections in PD

2. Page 3, line 66. Would “However” not make more sense to start the paragraph than “Nevertheless”? (because it is the finding of Desmurget, as well as the explanation of the “motor gate” hypothesis, that is now in contrast with then listed views for the basal ganglia's role in volitional vs. automatic movements?)

3. Although the reviewer #2 does not insist on necessarily adding power analysis that he originally requested and satisfied with your rebuttal, it may be helpful to consider including a statement to the treatment of this issue (along the lines that was presented in the rebuttal letter) to the body of the manuscript.

## Author Response

Dear Editor,

We would like to thank you and the reviewers for the opportunity to improve and revise our manuscript for consideration in eNeuro. We have revised the paper based on the positive feedback provided. Below, we respond to each of the comments.

Editor

1. Remove the Abbreviations paragraph/list. Please only use nonstandard abbreviations if a term appears three or more times (the initial appearance of the term and one other time). Spell out the term at first occurrence and introduce the abbreviation by placing it in parentheses after the term. Units should conform to the International System of Units (SI) (see the International System of Units Brochure), except for temperatures, which should be expressed in degrees Celsius.

-Thank you for bringing this to our attention. The abbreviation list has been removed.

2. The species studied is not mentioned in the abstract. Please make sure to update both the abstract in the article file and on the submission form

-Thank you for the suggestion. ‘Human participants’ have been specified in the abstract (Please see line 2).

3. The sex of the species studied is not mentioned in the materials and methods section. Please mention “male”, “female”, “x males and x females”, “of either sex”.

- Thank you for the opportunity to add these details. This has been specified in the methods (Please see lines 101 & 102).

Reviewers

1. Typo : page 1, line 10 “Eliminating this confound in a double-step task, we evaluated THE effect of conscious awareness of trajectory change on online motor corrections in PD

-Thank you for identifying this mistake. This typo has been corrected (Please see line 10).

2. Page 3, line 66. Would “However” not make more sense to start the paragraph than “Nevertheless”? (because it is the finding of Desmurget, as well as the explanation of the “motor gate” hypothesis, that is now in contrast with then listed views for the basal ganglia's role in volitional vs. automatic movements?)

- We appreciate this suggestion and have changed “nevertheless” to “however” (Please see line 64).

3. Although the reviewer #2 does not insist on necessarily adding power analysis that he originally requested and satisfied with your rebuttal, it may be helpful to consider including a statement to the treatment of this issue (along the lines that was presented in the rebuttal letter) to the body of the manuscript.

-Thank you for the opportunity to clarify this section of the manuscript. As per the reviewer's suggestion we have included a detailed explanation in our discussion (Please see lines 446-458).

We hope that we have addressed the minor issues raised by the reviewers and look forward to hearing from you.

Sincerely,

Kate Merritt, M.Sc.

The Brain and Mind Institute

University of Western Ontario

Dr. Penny A. MacDonald, MD, PhD, FRCP(C)

Department of Clinical Neurological Sciences

The Brain and Mind Institute

University of Western Ontario
